# HOMA-AD in Assessing Insulin Resistance in Lean Noncirrhotic HCV Outpatients

**DOI:** 10.1155/2012/576584

**Published:** 2012-07-16

**Authors:** Matheus Truccolo Michalczuk, Camila Rippol Kappel, Oscar Birkhan, Ana Carolina Bragança, Mário Reis Álvares-da-Silva

**Affiliations:** Gastroenterology Division, Hospital de Clínicas de Porto Alegre, Universidade Federal do Rio Grande do Sul, Ramiro Barcelos 2350, 90035-003 Porto Alegre, RS, Brazil

## Abstract

*Introduction*. There is an association between HCV and insulin resistance (IR), which is currently assessed by HOMA-IR. There is evidence that HOMA-adiponectin (HOMA-AD) is more accurate, but its role in HCV patients is unknown. The purpose of this study was to evaluate IR in an HCV sample and controls, in order to compare the accuracy of HOMA-IR and HOMA-AD. *Methods*. Ninety-four HCV outpatients aged <60 years who met the criteria of nondiabetic, nonobese, noncirrhotic, and nonalcohol abusers were included and compared to 29 controls. Fasting glucose, insulin, adiponectin, and lipid profiles were determined. IR was estimated by HOMA-IR and HOMA-AD. *Results*. The groups were similar regarding sex and BMI, but the HCV patients were older. The median insulin level was higher in the HCV group (8.6 mU/mL (6.5–13.7) versus 6.5 (4.3–10.7), *P* = 0.004), as was median HOMA-IR (1.94 (1.51 to 3.48) versus 1.40 (1.02 to 2.36), *P* = 0.002) and the prevalence of IR (38.3% versus 10.3% (*P* = 0.009)). No differences were found in adiponectin levels (*P* = 0.294) and HOMA-AD (*P* = 0.393). *Conclusion*. IR is highly prevalent even in low-risk HCV outpatients. Adiponectin is not influenced by the presence of HCV. HOMA-AD does not seem to be useful in assessing IR in HCV patients.

## 1. Introduction

The hepatitis C virus (HCV) is one of the most prevalent causes of chronic liver disease worldwide and leads to cirrhosis and hepatocellular carcinoma in a high percentage of carriers [1, 2].Insulin resistance (IR), impaired glucose tolerance, and type 2 diabetes mellitus (DM) are frequent extrahepatic manifestations [3, 4]. The mechanisms by which chronic HCV infection leads to IR remain uncertain, but it has been suggested that the blockade of IRS-1 and IRS-2 signaling via proinflammatory cytokines is involved [5, 6]. Conditions other than HCV are associated with IR, which include cirrhosis [4, 7], obesity [8], and advanced age [9]. These risk factors are often present in studies focusing on IR in HCV patients.

The adipocytokines (adiponectin, leptin, and resistin) are a family of adipose tissue-derived serum proteins that influence the glucose and lipid metabolism [10]. Adiponectin (AD) modulates hepatic fat content and acts as an insulin sensitizer and an anti-inflammatory and antifibrotic cytokine [11–15]. Nonalcoholic fatty liver disease (NAFLD), a model of IR, is related to low AD levels [16–18]. Some studies have investigated the role of AD in chronic hepatitis C, but it remains controversial. Some authors suggest that HCV increases AD levels [19–22], whereas others have not corroborated these results [23–30].

IR is evaluated by the homeostasis model assessment for insulin resistance (HOMA-IR), a surrogate of the hyperinsulinemic euglycemic clamp, which is considered the standard method in IR determination [31]. HOMA-AD was recently described for assessing IR. In a general Japanese study population, selected neither by body mass index (BMI) nor glucose profile, a good correlation between HOMA-AD and clamp was shown. In addition, HOMA-AD was more accurate than HOMA-IR, especially in patients with elevated fasting glucose levels and higher BMI [32].TheHOMA-AD index, that seems to be a reliable method in evaluating IR, has never been tested in patients with chronic hepatitis C. We hypothesized that patients with HCV, even without traditional risk factors for metabolic diseases, have a higher HOMA-IR and HOMA-AD than the control group.

## 2. Methods

### 2.1. Patients

 From July 2008 to December 2009, a total of 94 consecutive outpatients with chronic HCV infection, aged between 18 and 60 years were recruited at Hospital de Clínicas de Porto Alegre (HCPA). All subjects were seropositive for HCV antibody (confirmed by third-generation enzyme-linked immunosorbent assay (ELISA) and HCV RNA PCR). Ninety-two of them (97%) were genotyped, 73 (77%) had a available viral load, and 89 (95%) had undergone liver biopsy. The other 5 (5%) had no clinical signs of cirrhosis [normal prothrombin time and platelets levels, AST/platelets rate (APRI score <  0.5), and a normal ultrasonography]. Patients with concurrent liver disease caused by something other than HCV, alcohol abusers (>40 g/day), those infected by the human immunodeficiency virus (HIV) or those presenting severe cardiovascular disorder, chronic renal injury, pancreatitis, or evidence of malignant neoplasms, including hepatocellular carcinoma were excluded. Obesity (BMI >  30), DM, and previous or actual use of interferon, immunosuppressive or cholesterol-lowering drugs were additional exclusion criteria. The Human Research and Ethics committee approved the study, and informed consent was obtained from each patient.

During the same period, 29 healthy volunteers were selected as controls at the HCPA Blood Bank. All had negative serology for HIV and chronic viral hepatitis. They met the same exclusion criteria as the exposed population.

### 2.2. Anthropometric Assessment

Height, weight, and waist circumference were determined by a registered dietitian. BMI was calculated by the formula weight (kg)/height (m)^2^. 

### 2.3. Laboratory and Histopathological Assessment

Blood samples were obtained after a 12 h overnight fasting to determine plasma glucose, insulin, and lipid profiles (total cholesterol, HDL-cholesterol, and triglycerides) and for AD analysis. Glucose and lipid profile assessments were made with the enzymatic UV-hexokinase method in Advia 1800 equipment. The plasma insulin was measured by the electrochemiluminescence method with Modular equipment by Roche, and AD was measured using ELISA commercial kits according to the manufacturer's instructions (Adiponectin ELISA Kit, HU BioSource Europe S.A., Nivelles, Belgium).

Degree of insulin resistance was calculated according to the HOMA-IR using the following formula: fasting insulin level (mUI/L) × fasting glucose level (mg/dL)/405 [30]. According to the HOMA-AD index, it was calculated by the following formula: fasting insulin level (mUI/L) × fasting glucose level (mg/dL)/AD (mg/mL) [32]. A HOMA-IR index value of more than 2.71 was considered IR [33]. 

Ultrasound-guided liver biopsies were obtained and fragments were stained with hematoxylin-eosin. An expert pathologist assessed the biopsies according to the METAVIR scoring system. Fibrosis was staged on a scale from F0 to F4, listed as follows: F0: no fibrosis; F1: portal fibrosis, without septa; F2: few septa; F3: many septa without cirrhosis; F4: cirrhosis. Stages F0 and F1 were scored as minimal/mild fibrosis, and stages F2-F3 were scored as advanced fibrosis [34]. Patients in stage F3, if Ishak F5 (marked bridging fibrosis—incomplete cirrhosis), and those with METAVIR score of F4 (cirrhosis) were excluded from the study. 

HCV carriers were divided according to the genotype and viral load into two groups: genotype 3 or non-3 and high viral load (>400.000 UI/mL) or low viral load (<400.000 UI/mL), respectively. 

### 2.4. Statistical Analysis

Data were presented as mean ± standard deviation for variables with normal distribution and as median and interquartile range for asymmetric variables. The Student *t*-test or Mann-Whitney *U* test was used, depending on the distribution of the data (symmetrical or not) and homogeneity of variance. Categorical variables were compared by the Chi-square or Fisher's exact test. Correlations between HOMA-IR and HOMA-AD were assessed using Spearman's correlation coefficient analysis. All analyses were carried out using SPSS software version 11.0 (SPSS Inc., Chicago, IL, USA). All tests were two-tailed, and a *P* value of <0.05 was considered statistically significant.

## 3. Results

As shown in Table 1, regarding demographic characteristics and glucose and lipid profiles, the HCV patients were older, had lower total cholesterol, and higher insulin levels. There were no differences in gender, BMI, AD, HDL cholesterol, triglycerides or glucose levels between the groups.

Table 2 shows that HOMA-IR was significantly higher in the HCV group. Also, IR was more prevalent in the HCV patients. Conversely, no difference was found between the groups concerning the HOMA-AD index.

When evaluating the HCV patients' characteristics and their relation with IR, we found that patients with high viral load had a higher HOMA-IR when compared with those with low viral load. We could not find any influence of genotype or fibrosis on HOMA-IR (Table 3). 

**Figure 1:**
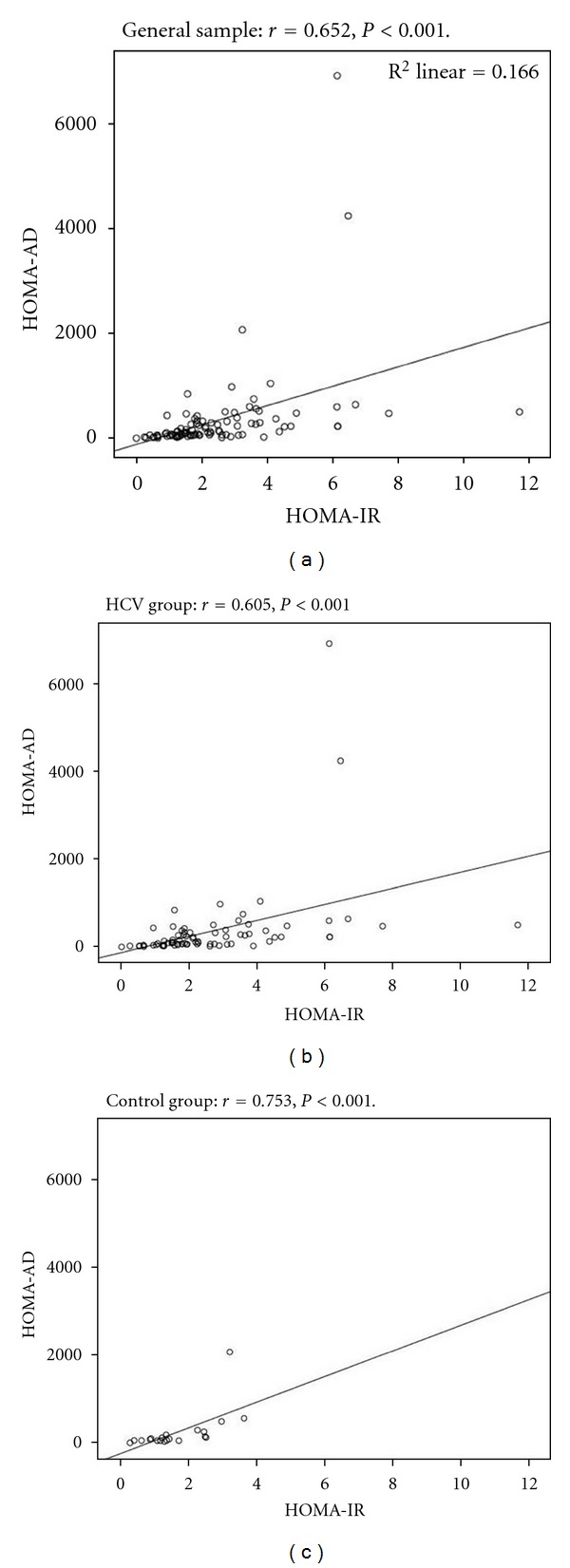
Spearman's correlation coefficient between HOMA-IR and HOMA-AD in the general sample, HCV, and control group.

HOMA-IR and HOMA-AD presented a strong correlation as assessed by Spearman's correlation coefficient analysis, varying between 0.652 in the general sample, 0.753 in noninfected individuals and 0.605 in HCV patients (*P* < 0.001 for all cases, as shown in Figure 1).

A logistic regression was performed to evaluate the influence of the possible confounding factors. IR was the main outcome. After the analysis, we found an odds ratio of 8.8 (1.4 to 54.9) for the association between IR and HCV. As shown in Table 4, the HCV group had higher HOMA-IR than controls, and this difference was still present after adjusting for total cholesterol, age, and BMI. The variables of total cholesterol and age did not correlate with HOMA-IR, whereas an increase of 1 kg/m^2^ in the BMI corresponds to an increase of 0.125 units in the natural logarithm of HOMA-IR.

## 4. Discussion

This study was designed to evaluate the relationship between HCV, IR, and AD in a selected group of individuals with no risk factors for metabolic diseases, such as obesity and DM. Also, patients with advanced liver disease, another condition known to be associated with IR, were excluded. 

AD plays a crucial role in glucose and lipid metabolism. Serum concentration of AD is inversely correlated with fat mass. Indeed, AD is downregulated in obesity and type 2 DM. The receptors involved in these processes are AdipoR1, expressed in skeletal muscle and other tissues, and AdipoR2, which is mostly localized in the liver [11–15]. Studies investigating the role of this cytokine in insulin sensitivity in HCV patients have yielded conflicting results (Table 5). These studies need to be interpreted with caution because some of them did not include a control group [19, 20, 24, 26] and some included patients with DM, cirrhosis, and obesity, the presence of which is considered to be confounding factors [19–21, 23, 24, 26–29]. Similar to the studies that excluded individuals with DM, obesity, and cirrhosis [22, 25], we evaluated IR by the HOMA-IR and HOMA-AD index and found differences in HOMA-IR between the groups; a result that was not achieved by Aksõz et al. [22] and Tanaka et al. [25]. Because there is strong evidence that HCV patients have more IR than controls [3, 4, 6, 21, 26, 35, 36] and the samples used by these authors were small (30 patients), the final IR and adiponectin levels could be biased. 

 The standard method in the IR evaluation is the hyperinsulinemic euglycemic clamp, but its cost and complexity limit its clinical applicability. HOMA-IR, the surrogate and most widely used method to assess IR, is cheap and easy to reproduce. Nevertheless, HOMA-IR has some limitations especially in patients with conditions such as impaired fasting glucose and/or high BMI. For this reason, we attempted to study the role of a new method of assessing IR: the AD-based HOMA-AD. Matsuhisa has demonstrated that HOMA-AD performs better than HOMA-IR in Japanese subjects with higher levels of glucose and elevated BMI, which suggests that HOMA-AD is promising [32]. To the best of our knowledge, HOMA-AD has never been tested in HCV patients.

HCV is associated with a specific metabolic syndrome, HCV-associated dysmetabolic syndrome, consisting of steatosis, hypocholesterolemia, and insulin resistance/diabetes. These metabolic derangements contribute to a decrease in sustained virological response (SVR) to pegylated-interferon-*α*-ribavirin as standard of care, and are associated with progression of liver fibrosis [37]. We found low cholesterol levels in HCV patients, as previously demonstrated [37–39]. In fact, HCV core protein reduces microsomal triglyceride transport protein function, leading to impairment of very low-density lipoprotein, triglyceride, and apolipoprotein B (APO-B) secretion, which in turn contributes to hepatic lipid accumulation and reductions in plasma total cholesterol [38].

HCV patients were compared to healthy controls. We need to address some issues regarding control group. They were younger than included HCV patients, a potential bias considering that older individuals are more prone to glucose metabolism disruptions. In order to thoroughly evaluate these differences, a multivariate analysis was performed, and the final results were not influenced by age, BMI, and total cholesterol. As a limitation to the analysis, we obtained a broad confidence interval, which can be explained by the small number of individuals in the control group with IR. Moreover, we did not rule out hepatic steatosis in control group, as they were not evaluated by ultrasound scanning. However, we have tried to minimize the possibility of NAFLD in this group, by selecting HCV negative, nonobese controls, all of them without metabolic syndrome, diabetes, or regular alcohol consumption. 

Our results demonstrate that HCV patients, despite the strict inclusion criteria, have higher HOMA-IR and a higher prevalence of IR than healthy individuals. Some authors have demonstrated the same results, but they did not use the same exclusion criteria, which may have led to a possible influence of factors, such as obesity, excessive alcohol consumption, cirrhosis, and DM on the final results [21, 22, 26, 35]. The specific mechanisms by which HCV leads to glucose metabolism disturbances are not fully understood, but it seems that increased insulin resistance is associated with oxidative stress and overproduction of inflammatory cytokines. The oxidative stress mediates signals involving the p38 mitogen-activated protein kinase and activates nuclear factor kappa B. This transcription factor plays a key role in the expression of cytokines, tumor necrosis factor alpha (TNF-alpha), interleukin 6, interleukin 8, tumor growth factor beta, and Fas ligand. TNF-alpha inhibits the function of insulin receptor substrates (IRS-1) and decreases the expression of the glucose transporter (GLUT-4) and lipoprotein lipase in peripheral tissues, which leads to impaired insulin action on peripheral tissues and hepatic glucose uptake [36, 40, 41]. We recently showed that HCV-naïve patients have a higher pro-anti-inflammatory cytokines ratio than controls [42]. However, when we evaluated IR and the correlation between adiponectin and IRS-1 (measured in liver and leucocytes) in a sample of lean, nondiabetic, noncirrhotic HCV patients compared to healthy controls, we found no relationship between leukocytes and liver IRS1 and adiponectin, although there was a significant decrease of IRS-1 in patients with HCV. This finding suggests that IR is unrelated to lipid metabolism in HCV patients [43].

We can highlight some studies on HCV and IR that shared similar aims and inclusion criteria with our study. For example, Moucari et al. [44] evaluated the association between IR (HOMA-IR >  3) and HCV (genotypes and viral load) and also hepatic fibrosis. The authors found IR in 32.4% of the HCV patients, whereas we found 38.3%. Moreover, they suggested that IR is specific to HCV, especially in genotype 1 patients with a high viral load and advanced fibrosis. Similarly, we found higher HOMA-IR in patients with high viral load, but we did not observe any differences in IR, genotype or liver fibrosis. In the present study, the number of patients with advanced fibrosis was too small (just 4 patients with F3, and none cirrhotic included) to allow any conclusions. Also, a *β*-error may have occurred. Another study by Vanni et al. [40] focused on the sites and mechanisms of IR (assessed by hyperinsulinemic euglycemic clamp) in 14 nonobese, nondiabetic patients with chronic HCV compared to 7 controls. The authors concluded that HCV itself is associated with peripheral and hepatic insulin resistance. They suggested that competition by increased lipid oxidation and possibly enhanced hepatic expression of inflammatory cytokines/mediators could be involved in the defective glucose regulation. Compared to these trials, we found similar results regarding the relationship of IR and HCV, but we demonstrated that HCV could exert this effect despite lipid metabolism.

In fact, we did not find differences between the groups regarding AD levels. The strict inclusion criteria (lean patients, no DM or cirrhosis) can partly explain these results. On the other hand, mechanisms of IR in HCV patients may be different from those related to other diseases, such as NAFLD. Regarding relation between hepatic steatosis and adiponectin in our sample, some individuals in the HCV group were not evaluated by US scanning and just a few (six patients) had hepatic steatosis at liver biopsy, which made it difficult to assess this relationship. When we considered the influence of AD on the IR assessed by HOMA-AD, no difference was observed between the HCV patients and individuals unexposed to the virus. The discrepancy between the results obtained by HOMA-IR and HOMA-AD can be explained by the absence of a direct effect of HCV on AD. It seems that AD does not influence IR in lean HCV patients. Finally, despite the fact that HOMA-IR and HOMA-AD showed a good correlation, the results they yielded were not concordant. This seems to have occurred because both indexes include glucose and insulin in their formula and AD has a minor influence on the mathematical correlation. 

In conclusion, HCV is related to IR even in the absence of obesity, DM, and cirrhosis, and it does not seem to be AD-dependent. Even though HOMA-AD is a promising method to assess IR in other conditions, it cannot be recommended for the evaluation of IR in the HCV population. We suggest that HCV patients, independent of risk factors for metabolic diseases, should be evaluated for IR. However, HOMA-IR cannot be replaced by HOMA-AD in the assessment of IR in HCV-infected patients.

## Figures and Tables

**Table 1:** Characteristics of the patients with chronic hepatitis C and controls.

	HCV	Controls	*P* value
	(*n* = 94)	(*n* = 29)	
Age (years)	46.9 (±8.1)	37.5 (±10.1)	<0.001
Gender (male)	47 (50.5%)	12 (41.4%)	0.516
Body mass index (kg/m^2^)	24.7 (±2.9)	25.4 (±2.8)	0.226
Glucose (mg/dL)	94.5 (±10.2)	90.6 (±9.8)	0.070
Insulin (mU/mL)	8.6 (6.5 to 13.7)	6.5 (4.3 to 10.7)	0.004
Adiponectin (mg/mL)	6.5 (3.5 to 13.0)	4.6 (3.1 to 8.8)	0.294
Total cholesterol (mg/dL)	163.4 (±32.6)	206.4 (±43.9)	<0.001
HDL cholesterol (mg/dL)	51.9 (±15.7)	55.9 (±17.2)	0.247
Triglycerides (mg/dL)	94.5 (67.5 to 119.2)	121.0 (75.0 to 170.5)	0.059

Quantitative variables described by mean ± sd and compared by the Student *t*-test for independent samples or by median and interquartile range and compared by the Mann-Whitney *U* test, depending on the distribution of the data (symmetrical or not). Categorical variables described by *n* (%) and compared by the Chi-square or Fisher exact test.

**Table 2:** IR profile in HCV and non-HCV individuals according to HOMA-IR and HOMA-AD.

	HCV	Non-HCV	*P* value
	(*n* = 94)	(*n* = 29)	
HOMA-IR	1.9 (1.5 to 3.5)	1.4 (1.0–2.4)	0.002
IR	36 (38.3%)	3 (10.3%)	0.009
HOMA-AD	128.9 (59.1 to 369.2)	96.7 (56.7 to 240.0)	0.459

^∗^IR defined as HOMA-IR > 2.7. Quantitative variables described by median and interquartile range and compared by the Mann-Whitney *U* test. Categorical variables described by *n* (%) and compared by Chi-square or Fisher exact test.

**Table 3:** HOMA-IR according to viral characteristics of patients with chronic hepatitis C.

Variables	*N*	HOMA-IR	*P*
Genotype	92		
Non-3	74 (80.4%)	1.94 (1.51–3.61)	0.890
3	18 (19.6%)	1.93 (1.46–3.56)	
Fibrosis stage^∗^	89		
F0/F1	63 (70.8%)	1.93 (1.51–2.93)	0.207
F2/F3	26 (29.2%)	2.75 (1.55–3.95)	
Viral load	73		
>400.000 UI/mL	55 (75.3%)	1.53 (1.17–2.35)	0.027
<400.000 UI/mL	18 (24.7%)	2.21 (1.29–3.76)	

^∗^METAVIR.

**Table 4:** Influence of total cholesterol, age, and BMI in the results of the HOMA-IR natural logarithm in HCV and non-HCV individuals: a multiple linear regression.

	**B (CI 95%)	^ ∗∗∗^ *β*	*P*
HCV/non-HCV	0.39 (0.23–0.75)	0.23	0.036
Total cholesterol	0 (−0.004–0.003)	−0.009	0.925
Age	0.007 (−0.007–0.021)	0.097	0.311
BMI	0.125 (0.083–0.168)	0.496	<0.001

*R ^2^ = 0.31. Dependent quantitative variable: natural logarithm of HOMA-IR.

**B: Unstandardized coefficients (95% confidence interval).

^∗∗∗^ *β*: Standardized *β* coefficient.

**Table 5:** Main results of the studies with reference to HCV, IR, and adiponectin.

Author, year, ref	HCV (*n*)	Controls (*n*)	Confounding factors (%)	HOMA-IR	Adiponectin
				(HCV versus controls)	(HCV versus controls)
	11		Cirrhosis (0)	No information	Similar
Lu et al., 2005 [23]	10 HBV	21	DM (0)		
			Obesity—no information		

	30	30	Cirrhosis (0)	Similar	Similar
Aksõz et al., 2008 [22]			DM (0)		
			Obesity (0)		

	30	30 HBV	Cirrhosis (0)	Similar	Similar
Tanaka et al., 2008 [25]			DM (0)		
			Obesity (0)		

	154	75	Cirrhosis (0)	Higher in HCV	Similar
Cua et al., 2007 [26]			DM (0)		
			Obesity—no information		

	51	24	Cirrhosis (0)	No information	Similar
Tiftikci et al., 2009 [27]			DM—no information		
			Obesity—no information		

Zografos et al., 2008 [28]	83	43	No information	No information	No information

	81	40	Cirrhosis (34)	Higher in HCV	Higher in HCV
Hung et al., 2009 [21]			F3/F4		
			DM (0)		
			Obesity—no information		

	94	29	Cirrhosis (0)	Higher in HCV	Higher in HCV
Present study/2011			DM (0)		
			Obesity (0)		
